# Network Topological Analysis for the Identification of Novel Hubs in Plant Nutrition

**DOI:** 10.3389/fpls.2021.629013

**Published:** 2021-02-10

**Authors:** Dario Di Silvestre, Gianpiero Vigani, Pierluigi Mauri, Sereen Hammadi, Piero Morandini, Irene Murgia

**Affiliations:** ^1^Proteomic and Metabolomic Laboratory, ITB-CNR, Segrate, Italy; ^2^Plant Physiology Unit, Department of Life Sciences and Systems Biology, University of Turin, Turin, Italy; ^3^Department of Environmental Science and Policy, University of Milan, Milan, Italy; ^4^Department of Biosciences, University of Milan, Milan, Italy

**Keywords:** *Arabidopsis thaliana*, co-expression, *Cucumis sativus*, iron, molybdenum, network topology, protein–protein interaction, plant nutrition

## Abstract

Network analysis is a systems biology-oriented approach based on graph theory that has been recently adopted in various fields of life sciences. Starting from mitochondrial proteomes purified from roots of *Cucumis sativus* plants grown under single or combined iron (Fe) and molybdenum (Mo) starvation, we reconstructed and analyzed at the topological level the protein–protein interaction (PPI) and co-expression networks. Besides formate dehydrogenase (FDH), already known to be involved in Fe and Mo nutrition, other potential mitochondrial hubs of Fe and Mo homeostasis could be identified, such as the voltage-dependent anion channel VDAC4, the beta-cyanoalanine synthase/cysteine synthase CYSC1, the aldehyde dehydrogenase ALDH2B7, and the fumaryl acetoacetate hydrolase. Network topological analysis, applied to plant proteomes profiled in different single or combined nutritional conditions, can therefore assist in identifying novel players involved in multiple homeostatic interactions.

## Introduction

Living organisms are increasingly viewed as integrated and communicating molecular networks, thanks also to the diffusion of data-derived Systems Biology approaches (Barabási and Oltvai, 2004). Such approaches are well established in biomedical and pharmaceutical research (Zhou et al., 2014; Guney et al., 2016) but not widely used in plant science. However, a growing number of plant biologists are adopting them, also to achieve a refined comprehension of plant nutrition (Mai and Bauer, 2016; Di Silvestre et al., 2018; Tsai and Schmidt, 2020).

Plant systems biology studies are dominated by transcriptomic data and statistics that, by measuring the dependence between variables (transcripts), allow us to reconstruct and analyze co-expression network models (Rao and Dixon, 2019). Instead, fewer studies rely on protein–protein interaction (PPI) networks, mainly due to the lack of accurate plant models (Di Silvestre et al., 2018). Nevertheless, the number of studies focusing on high-throughput profiling of plant proteomes (Vigani et al., 2017; Castano-Duque et al., 2018; Santos et al., 2019), on experimental identification of PPIs (Senkler et al., 2017; Osman et al., 2018; Huo et al., 2020) or their computational prediction (Ding and Kihara, 2019) are recently increasing. The computational prediction of PPI is usually inferred by transferring interactions from model plant orthologs, like *Arabidopsis thaliana.* This approach represents one of the most important resources for network inference in plant organisms, with some flaws, including the identification of false positives and inadequate coverage from associalogs (Lee et al., 2015).

The co-expression networks reconstructed from proteomic data may represent an alternative to the lack of accurate PPI models and a tool for handling, at the system level, large-scale proteomic datasets related to the non-model plant. Similar to transcript networks, protein co-expression networks are defined as an undirected graph where nodes correspond to proteins and edges indicate significant correlation scores. By exploiting the laws underlying graph theory, the identification of hubs/bottlenecks and of differentially correlated proteins are sought, as well as the topological and functional modules related to specific biological phenotypes (Vella et al., 2017).

The studies that elucidate plant iron (Fe) nutrition are steadily increasing due to their impact on alleviating nutritional deficiencies in humans (Murgia et al., 2012, 2013).

Given these premises, we performed the topological analysis of PPI and protein co-expression networks to identify new proteins involved in Fe and molybdenum (Mo) homeostasis in plants by taking into account subcellular compartmentalization. In particular, we reconstructed and analyzed networks from mitochondrial proteomes purified from roots of *Cucumis sativus* plants grown under single or combined Fe and Mo starvation (Vigani et al., 2017).

## Materials and Methods

### Reconstruction of *C. sativus* Protein–Protein Interaction (PPI) Networks

*A C. sativus* PPI network was reconstructed based on homology with *A. thaliana* by using the STRING v11 database (Szklarczyk et al., 2019). Retrieved homologous interactions were filtered by retaining exclusively those databases annotated and/or experimentally determined with a STRING Score of >0.15 and >0.35, respectively. Following these parameters, a fully connected network of 903 nodes and 10456 edges was built. Four different sub-networks were then extracted by considering the proteins identified in each condition analyzed: +Mo+Fe, 463 nodes and 4118 edges; −Mo+Fe, 466 nodes and 4064 edges; +Mo−Fe, 480 nodes and 4682 edges; and −Mo−Fe, 474 nodes and 4425 edges.

### Functional Modules in *C. sativus* PPI Network

A set of proteins differentially expressed in *C. sativus* root mitochondria under different conditions (+Mo+Fe, +Mo−Fe, −Mo+Fe, and −Mo−Fe) were selected by Linear Discriminant Analysis (LDA, *P* < 0.05 and F ratio > 3), as previously reported (Vigani et al., 2017). Starting from this set of differentially expressed proteins, a *C. sativus* PPI network was reconstructed by homology with *A. thaliana* using STRING v11 database, as reported above. Proteins were grouped in functional modules by the support of BINGO (Maere et al., 2005) and STRING (Doncheva et al., 2019). Cytoscape’s Apps; the network was visualized by the Cytoscape platform, and node color code indicates upregulated (red) and downregulated (light blue) proteins based on *Spectral count* (SpC) normalization (SpC normalized in the range 0–100, by setting to 100 the higher SpC value per protein).

### Reconstruction of *C. sativus* Protein Co-expression Networks

Protein co-expression networks were reconstructed by processing *C. sativus* root mitochondria protein profiles characterized under different conditions: +Fe, *n* = 12; +Mo, *n* = 14, −Fe, *n* = 15; −Mo, *n* = 13. Only proteins identified at least in 51% of the considered samples were retained and processed for each condition. Protein data matrices were processed using *Spearman*’s rank correlation coefficient; *Spearman*’s rank correlation score >|0.7| and *P* < 0.01 were set as thresholds. All processing was performed using the statistical software JMP15.2, while networks were visualized and analyzed by the Cytoscape platform and its plugins (Su et al., 2014).

### Topological Analysis of *C. sativus* PPI and Protein Co-expression Networks

Both PPI and co-expression networks were topologically analyzed by Centiscape Cytoscape’s App (Scardoni et al., 2014). As for PPI networks, *Betweenness* and *Centroid* centralities were calculated, and nodes with values above the average calculated on the whole network were considered hubs (Di Silvestre et al., 2018). On the contrary, a set of differentially correlated proteins were selected in the co-expression network by filtering their *Degree* centrality.

## Results and Discussion

### PPI Networks

The analysis of root mitochondria of *C. sativus* plants grown under Mo and/or Fe starvation led to the identification of 1419 proteins (Vigani et al., 2017). Starting from this proteome, a *C. sativus* PPI network, connecting 903 nodes through 10464 edges, was built by exploiting the interactions between orthologs in *A. thaliana;* the average homology between *C. sativus* and *A. thaliana* was around 67% (Supplementary Figure 1). A sub-network made of 118 nodes and 388 interactions was extracted by considering proteins (*n* = 134) whose level was significantly altered in at least one of the four nutritional conditions (control, single or combined Mo and Fe starvation) (Vigani et al., 2017). Proteins belonging to this sub-network were grouped in 21 functional modules, including redox homeostasis, amino acid metabolism, aldehyde dehydrogenases, protein folding, and electron transport chain (Supplementary Figure 2). The analysis of the behavior of their members under the various nutritional conditions indicated above can support the identification of novel candidate genes; in this paragraph, we will highlight some interesting examples.

Fe starvation, combined with Mo starvation or sufficiency, upregulates several proteins involved in amino acid metabolism, such as glutamate dehydrogenases GDH1 and GDH2, aminobutyric acid transaminase POP2 involved in the metabolism of γ-amino butyrate (GABA), which links C and N metabolism in mitochondria (Shelp et al., 2012), aspartate aminotransferase ASP1, the two subunits MCCA and MCCB of methyl crotonyl-CoA carboxylase involved in leucine catabolism, and beta-cyanoalanine synthase/cysteine synthase CYSC1, which detoxifies the cyanide produced as by-product of ethylene synthesis (Hatzfeld et al., 2000; Yamaguchi et al., 2000; Garcia et al., 2010; Arenas-Alfonseca et al., 2018; Supplementary Figure 2). Notably, ethylene is a well-established regulator of Fe deficiency responses (Romera et al., 1999; Lucena et al., 2015). An increased concentration of amino acids has been documented in the xylem sap of Fe deficient cucumber plants (Borlotti et al., 2012). These results suggest that, under Fe starvation, a larger part of the amino acid metabolism takes place in the radical apparatus, with respect to the aerial parts of the plants.

The redox homeostasis group includes formate dehydrogenase (FDH), the enzyme catalyzing the oxidation of formate into CO_2_ (Alekseeva et al., 2011) and involved in Fe and Mo homeostasis (Vigani et al., 2017; Murgia et al., 2020). This group also includes various proteins upregulated under Fe starvation, such as glutathione peroxidase GPX6, alternative oxidase AOX2, glyoxalase II3/sulfur dioxygenase GLY3, At5g42150 coding for another glutathione peroxidase and aldehyde dehydrogenase ALDH2B7 (Supplementary Figure 2). The aldehyde dehydrogenase enzymes can act as “aldehyde scavengers” of reactive aldehydes produced during the oxidative degradation of membrane lipids (Brocker et al., 2013). In particular, ALDH2B7 converts acetaldehyde into acetic acid (Rasheed et al., 2018). The redox homeostasis group also includes SAG21, which is strongly upregulated under single Mo starvation (Supplementary Figure 2). Perturbation of SAG21 expression affects biomass, flowering, the onset of leaf senescence, the pathogens’ ability to proliferate (Salleh et al., 2012) and alters the function or stability of mitochondrial proteins involved in ROS production and/or signaling (Salleh et al., 2012).

The fatty acid metabolism group includes dihydrolipoamide branched chain acyltransferase BCE2, also known as Dark Inducible 3 (DIN3), whose expression increases soon after exposure to darkness (Fujiki et al., 2000, 2005). The perturbing effects of prolonged darkness on cellular Fe status and its impact on the expression of Fe homeostasis genes are well known: as an example, the light/dark circadian cycles as well as prolonged darkness influence the expression of the iron-storage ferritin, whose gene expression is activated by prolonged darkness (Tarantino et al., 2003) and is circadian-regulated (Duc et al., 2009; Hong et al., 2013). BCE2 is upregulated under Fe starvation (Supplementary Figure 2); this finding is intriguing since darkness is associated with a rise in free Fe ions due to the dismantling of the photosynthetic structures. It would be interesting to investigate BCE2 regulation under Fe excess to establish if BCE2 is upregulated by Fe starvation only or, more generally, by Fe fluctuations.

The protein folding group includes the brassinosteroid (BR) biosynthesis gene DWF1, which mediates BR biosynthesis during the positive growth responses of the root system to low nitrogen (Jia et al., 2020). DWF1 is downregulated under Fe starvation and upregulated under single Mo starvation (Supplementary Figure 2).

The cell cycle/division group includes the mitochondrial GTPase MIRO1 (Supplementary Figure 2), which regulates mitochondrial trafficking and shape in eukaryotic cells. In particular, MIRO1 GTPase influences mitochondrial morphology in *A. thaliana* pollen tubes (Yamaoka and Leaver, 2008; Sørmo et al., 2011); interacting partners of MIRO GTPases are sought to better elucidate their functions in mitochondrial dynamics (Yamaoka and Hara-Nishimura, 2014). Notably, MIRO1 is upregulated under single Fe or Mo starvation but not under combined deficiencies (Supplementary Figure 2).

The histone/chromatin group includes GYRB2, a DNA topoisomerase (Wall et al., 2004) upregulated under Fe starvation (Supplementary Figure 2).

The translation group includes the pentatricopeptide protein PPR336, which associates with mitochondrial ribosomes (Waltz et al., 2019); PPR336 is downregulated under single Mo starvation, whereas it is upregulated under double Fe and Mo starvation (Supplementary Figure 2).

The electron transport chain group includes the mitochondrial carrier UCP1/PUMP1 transporting aspartate_*out*_/glutamate_*in*_ (as well as other amino acids, dicarboxylates, phosphate, sulfate, and thiosulfate) across the mitochondrial membrane (Monne et al., 2018); such carrier is upregulated in the single or combined Fe or Mo starvation (Supplementary Figure 2).

Transmembrane transporter activity group includes OM47 related to the voltage-dependent anion channel VDAC family; members of this family are major components of the outer mitochondrial membrane and are involved in the channeling of the products of chloroplast breakdown into the mitochondrion and in the exchange of various compounds between the cytosol and the mitochondrial intermembrane space (Li et al., 2016). OM47 is upregulated under single Fe starvation (Supplementary Figure 2). The role of VDAC protein family also emerged by the topological analysis of PPI network. Indeed, 91 PPI hubs were selected in these conditions: 27 hubs in control (+Mo+Fe), 17 hubs in +Mo−Fe, 26 hubs in −Mo+Fe, and 21 hubs in −Mo−Fe (Supplementary Table 1), while 28 PPI hubs were specifically related to sufficiency or starvation of either Fe or Mo (5 hubs in +Fe, 10 hubs in −Fe, 2 hubs in +Mo, and 11 hubs in −Mo) (Supplementary Table 2). In this scenario, VDAC4 and VDAC2 turned out as hubs in PPI networks under Mo starvation (Supplementary Table 2).

The proteins downregulated under Fe or Mo starvation can represent interesting homeostatic regulators in the PPI networks, and their occurrence should not be neglected in future, more extensive analyses; as an example, rice OsIRO3 plays an important role in the Fe deficiency response by negatively regulating the *OsNAS3* expression and, thus, nicotianamine NA levels (Wang et al., 2020).

### Co-expression Networks

**FIGURE 1:**
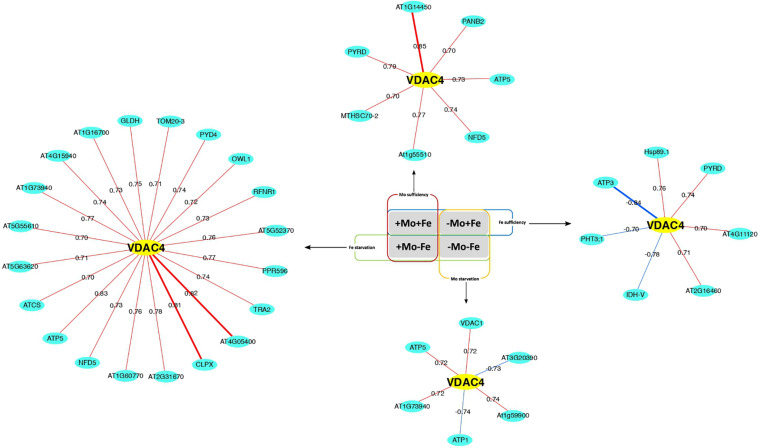
Differential VDAC4 correlation degree in co-expression networks. *Cucumis sativus* root mitochondria proteins identified under different conditions (+Mo+Fe, +Mo–Fe, –Mo+Fe, and –Mo–Fe) were analyzed according to their grouping into Fe sufficiency, Mo sufficiency, Fe starvation, and Mo starvation. Red and blue edges indicated positive and negative correlations, respectively. Edges width is proportional to *Spearman*’s correlation score (*P* < 0.01).

Co-expression networks were reconstructed from proteomes of purified mitochondria of *C. sativus* plants grown under different Mo and Fe nutritional conditions, reported in Vigani et al. (2017). In particular, the networks were reconstructed under Mo sufficiency (+Mo+Fe and +Mo−Fe conditions, 252 nodes and 1634 edges), Fe sufficiency (+Mo+Fe and −Mo+Fe conditions, 248 nodes and 2193 edges), Fe starvation (+Mo−Fe and −Mo−Fe conditions, 246 nodes and 1911 edges), and Mo starvation (−Mo+Fe and −Mo−Fe conditions, 240 nodes and 1547 edges). Around 5 to 7% of the proteins that were retrieved as co-expressed were also physically interacting (Supplementary Table 3). Following the network topological analysis, proteins belonging to the bioenergetics and amino acids metabolisms were found topologically relevant under Fe sufficiency (18 protein hubs) (Table 1A) or Mo sufficiency (12 protein hubs) (Table 1B). Moreover, 15 proteins emerged as network hubs under Fe starvation (Table 1C) and 16 proteins as hubs under Mo starvation (Table 1D); as examples, we then reconstructed the interactions for three hubs under Mo starvation, i.e. FDH (Table 1D and Supplementary Figure 3), ALDH2B7 (Table 1D and Supplementary Figure 4), CYSC1 (Table 1D and Supplementary Figure 5), and two hubs under Fe starvation, the Voltage-Dependent Ion Channel VDAC4 (Table 1C and Figure 1) and fumaryl acetoacetate hydrolase At4g15940 (Table 1C and Supplementary Figure 6).

**TABLE 1:** Proteins differentially correlated in *Cucumis sativus* co-expression networks (Fe sufficiency, Mo sufficiency, Fe starvation, and Mo starvation).

	Annotation					LDA		Degree				Function
	UNIPROT ID (*C. sativus)*	Gene name *C. sativus*	Gene name *A. thaliana*	Homology	Score	F Ratio	Prob > F	+Fe	+Mo	−Fe	−Mo	
**(A)**	A0A0A0K4Q8	Csa_7G073600	MCCB (AT4G34030)	77%	918	11,1	1,1E-04	**55**	5	2	9	Methylcrotonyl-CoA carboxylase, subunit beta
	A0A0A0LM23	Csa_2G382440	FLOT1 (AT5G25250)	75%	723	4,6	1,2E-02	**51**				Flotilins-like
	A0A0A0LXD4	Csa_1G424875	AT4G30010	69%	142			**42**	1	2	8	ATP-dependent RNA helicase
	A0A0A0L0B9	Csa_4G082380	MBL1 (AT1G78850)	50%	434	3,9	2,1E-02	**39**				Mannose-binding lectin
	A0A0A0K5L0	Csa_7G387180	MCCA (AT1G03090)	69%	1023			**38**	4	3	10	Methyl crotonyl-CoA carboxylase subunit alpha
	A0A0A0KJ29	Csa_6G526470	AT3G58140	74%	657			**36**				Phenylalanyl-tRNA synthetase
	A0A0A0KCX8	Csa_6G077980	ARGAH1 (AT4G08900)	85%	590			**34**	1	0	4	Arginase
	A0A0A0LBW6	Csa_3G199630	EDA9 (AT4G34200)	83%	981	4	1,9E-02	**33**	7			D-3-phosphoglycerate dehydrogenase
	A0A0A0L0I0	Csa_4G285780	PA2 (AT5G06720)	53%	335	3,7	2,6E-02	**33**	3	1	3	Peroxidase
	A0A0A0KA81	Csa_6G088110	AT2G20420	88%	757			**31**	4	8	12	Succinyl-CoA ligase
	A0A0A0LBB3	Csa_3G760530	SVL1 (AT5G55480)	56%	851	6,6	2,3E-03	**27**				Glycerophosphoryl diester- phosphodiesterase
	A0A0A0LFK3	Csa_3G734240	TIM9 (AT3G46560)	82%	162			**26**	0	2	1	Translocase of the inner membrane 9
	A0A0A0KRD9	Csa_5G613510	UOX (AT2G26230)	68%	434			**25**				Urate oxidase
	A0A0A0L542_ A0A0A0LXV7	Csa_3G078260 Csa_1G660150	GPT2 (AT1G61800)	75%	568	5	8,4E-03	**25**				Glucose-6-phosphate/phosphate translocator
	A0A0A0K1S9_ A0A0A0K3R7	Csa_7G047450 Csa_7G047440	AT2G20710	45%	415			**24**	8	3	1	PPR-type organelle RNA editing factor
	A0A0A0LS81	Csa_1G096620	ASP1 (AT2G30970)	88%	768			**22**	1	11	0	Aspartate transaminase
	A0A0A0LQ27	Csa_1G025890	OAT (AT5G46180)	79%	738			**21**	7	3		Ornithine delta aminotransferase
	A0A0A0L404	Csa_4G646110	FTSH4 (AT2G26140)	81%	1128			**19**	2	1	1	ATP-dependent zinc metalloprotease

**(B)**												

	A0A0A0KIM0	Csa_6G497010	NFS1 (AT5G65720)	78%	756				**33**	2		Cysteine desulfurase
	A0A0A0KB82	Csa_7G407690	AT5G61310	67%	95,1			4	**27**			Cytochrome c oxidase subunit
	A0A0A0L3T5	Csa_4G642530	VDAC2 (AT5G67500)	53%	317				**26**			Voltage-gated anion channel
	A0A0A0KMP0	Csa_5G321480	AT2G07698	93%	562			12	**22**	15	1	ATP synthase subunit alpha
	A0A0A0LGF5	Csa_2G033990	LON1 (AT5G26860)	75%	1456			10	**22**	9	3	ATP-dependent serine protease
	A0A0A0KW78	Csa_4G017120	TOM40-1 (AT3G20000)	71%	472			9	**22**	10	4	Component of mitochondrial outer membrane translocase
	A0A0A0LXK1	Csa_1G629760	SDH6 (AT1G08480)	68%	144			8	**22**	13	3	Component of succinate dehydrogenase complex
	A0A0A0KGU5	Csa_6G500700	MIC60 (AT4G39690)	36%	176			17	**19**	11	8	Component of mitochondrial transmembrane lipoprotein complex
	A0A0A0KGW6	Csa_6G366300	COS1 (AT2G44050)	66%	263	18,2	2,8E-06		**16**	5		6,7-Dimethyl-8-ribityllumazine synthase
	A0A0A0KMM1	Csa_5G047770	AT1G14930	36%	110			17	**15**			Bet v1-type pathogenesis-related protein
	A0A0A0K9E8	Csa_6G046410	SD3 (AT4G00026)	60%	297			3	**15**	2	0	Mitochondrial translocase
	A0A0A0LSR2	Csa_1G024260	AT3G18240	62%	506				**14**	13	9	Mitochondrial ribosomal subunit

**(C)**												

	A0A0A0KKV4	Csa_6G525450	RFNR1 (AT4G05390)	78%	630			8	2	**52**		Ferredoxin-NADP + reductase
	A0A0A0LDA3	Csa_3G435020	MPPa1 (AT1G51980)	63%	616	7,4	1,2E-03	13	6	**49**	12	Subunit alpha of mitochondrial processing peptidase complex
	A0A0A0L7Y6	Csa_3G164480	AT4G15940	76%	347					**48**		Fumaryl acetoacetate hydrolase
	A0A0A0KH76	Csa_6G188090	AT5G52370	57%	151					**44**		Mitochondrial 28S ribosomal protein S34
	E1B2J6	GAPDH	GAPC1	88%	610				9	**35**		Glyceraldehyde 3 phosphate dehydrogenase
	A0A0A0KBL8	Csa_6G077460	TKL2 (AT2G45290)	83%	1306	6,8	1,9E-03	0	5	**32**	5	Transketolase
	A0A0A0KRJ5	Csa_5G168830	MKP11 (AT5G17165)	51%	89	9,7	2,4E-04			**29**		Late embryogenesis abundant protein
	A0A0A0M1R9_ A0A0A0LKR1	Csa_1G574970 Csa_2G000830	HXK1 (AT4G29130)	74%	757			11	4	**27**	3	Hexokinase
	A0A0A0KXY5	Csa_4G337910	AT5G63620	84%	288			4	3	**27**	2	Zinc-dependent alcohol dehydrogenase
	A0A0A0LKD3	Csa_2G346040	UGP1 (AT5G17310)	51%	367			13	2	**27**	11	UDP-glucose pyrophosphorylase
	A0A0A0L2N5	Csa_4G310720	FAC1 (AT2G38280)	80%	1368	5,4	6,0E-03			**22**	11	AMP deaminase
	A0A0A0LZS4	Csa_1G423090	VDAC4 (AT5G57490)	63%	367			7	7	**20**	6	Voltage-gated anion channel
	A0A0A0LYA6	Csa_1G532350	AT4G33070	81%	1042			3	6	**19**	5	Thiamine pyrophosphate dependent pyruvate decarboxylase
	A0A0A0LQ20	Csa_2G403690	CoxX3 (AT1G72020)	66%	138			16	12	**17**	9	TonB-dependent heme receptor A
	A0A0A0KGH2	Csa_6G502730	AT2G18330	75%	891					**17**	2	ATPase

**(D)**												

	A0A0A0LLE7_ A0A0A0KLP6	Csa_2G372170 Csa_6G497310	SHM1 (AT4G37930)	80%	869			9	2	10	**28**	Serine hydroxymethyl transferase
	A0A0A0KGA1	Csa_6G135470	ALDH5F1 (AT1G79440)	77%	813	3,5	3,0E-02	7	3	9	**20**	Succinate-semialdehyde dehydrogenase
	A0A0A0L5J6	Csa_3G115030	AT5G40810	87%	514	3,4	3,6E-02	6	8	5	**18**	Cytochrome c1 component of cyt-bc1 complex
	A0A0A0LP60	Csa_2G360050	SDH5 (AT1G47420)	56%	266			4	4	4	**16**	Succinate dehydrogenase subunit 5
	A0A0A0KP30	Csa_5G199270	PIP1;4 (AT4G00430)	87%	523	15,4	1,0E-05	10	11	3	**19**	Aquaporin
	A0A0A0LJB4	Csa_2G010420	ALDH2B7 (AT1G23800)	80%	888	18,3	2,7E-06	0	7	3	**21**	Aldehyde dehydrogenase
	A0A0A0KYN6	Csa_4G192110	GDH1 (AT5G18170)	91%	788	24,1	2,7E-07	9	11	2	**20**	Glutamate dehydrogenase
	A0A0A0KWX8	Csa_4G050830	PGD1 (AT1G64190)	87%	892	15,9	8,2E-06	3	4	1	**24**	Phosphogluconate dehydrogenase
	A0A0A0KX20	Csa_4G052590	NDPK4 (AT4G23900)	78%	387	4,8	9,4E-03	15	2	1	**14**	Nucleoside diphosphate kinase
	A0A0A0LHY6	Csa_3G836500	FDH (AT5G14780)	83%	628	15,4	1,0E-05	2	2	1	**22**	Formate dehydrogenase
	A0A0A0K9Z6	Csa_6G004600	CYSC1 (AT3G61440)	79%	588	28,4	6,5E-08	8	11	0	**16**	b-cyanoalanine synthase/cysteine synthase
	A0A0A0KKD9	Csa_5G149330	BIP2 (AT5G42020)	91%	1229	3,3	3,7E-02	14			**20**	Heat shock protein 70
	A0A0A0K7B3	Csa_7G222870	CYS4 (AT4G16500)	35%	96	10,2	1,9E-04	5			**22**	Cysteine-type endopeptidase inhibitor
	A0A0A0LYF4	Csa_1G710160									**33**	Cysteine-type endopeptidase inhibitor
**(D)**	A0A0A0KVK8 A0A0A0LRR3_ A0A0A0LDZ4	Csa_4G094520 Csa_2G369070 Csa_3G889810	GF14 (AT1G35160)	93%	482						**33**	14-3-3 protein
	A0A0A0K6A8	Csa_7G070770	TUA4 (AT1G04820)	98%	869	3,5	3,3E-02				**17**	Tubulin

*Protein selection was based on the differential node Degree: Proteins with a Degree above the Network Average Degree (in bold) were retained. For each selected C. sativus protein, the C. sativus gene name, the name of A. thaliana homologous gene and the corresponding homology (in percent value and score), Linear Discriminant Analysis (LDA) parameters (only for differentially expressed proteins), and node Degree in the various nutritional conditions are reported. Homologies were retrieved by STRING database. Co-expression hubs in **(A)** Fe sufficiency (+Mo+Fe, −Mo+Fe), **(B)** Mo sufficiency (+Mo+Fe, +Mo−Fe), **(C)** Fe starvation (+Mo−Fe, −Mo−Fe) and **(D)** Mo starvation (−Mo+Fe, −Mo−Fe) are shown. Each accession number within brackets refers to the A. thaliana gene reported on the upper line.*

Formate dehydrogenase has been associated with stress responses in plants (Hourton-Cabassa et al., 1998; Shiraishi et al., 2000; Herman et al., 2002; Lou et al., 2016; Murgia et al., 2020), and it takes part in Fe and Mo homeostasis (Vigani et al., 2017; Murgia et al., 2020).

While FDH is co-expressed with a low number of proteins under Fe sufficiency, Fe starvation, and Mo sufficiency, such number strikingly increases under Mo starvation. Indeed, under this condition, FDH is co-expressed with 22 proteins (Table 1); some of these 22 proteins belong to amino acid/protein metabolisms (e.g., NADH dehydrogenase ubiquinone 1 encoded by AT3G08610; aspartic-type endopeptidase APF1; mitochondrial peptidase (MPPBETA) or redox activity (ALDH5F1, ALDH2B7, ALDH6B2; POP2; BCE2) (Supplementary Figure 3).

ALDH2B7 and CYSC1 are themselves hubs under Mo starvation (Table 1); indeed, under this condition, 21 different proteins are co-expressed with ALDH2B7, whereas, under the other conditions, a total of 10 proteins are co-expressed with ALDH2B7 (Table 1). The majority of these are involved in the synthesis/utilization of acetyl CoA metabolic intermediate, such as (i) MCCB (3-methylcrotonyl-CoA carboxylase) involved in the branched chain amino acids metabolism and (ii) ATCS (citrate synthase) and SDH1-1 (succinate dehydrogenase) involved in TCA cycle (Supplementary Figure 4). The modulation of ALDH2B7 expression observed under stress might play a role in the aerobic detoxification of acetaldehyde in plants (Rasheed et al., 2018). The presence of several co-expressed proteins involved in amino acids and GABA metabolisms (GDH1, POP2), in formate metabolism (FDH), and in the nucleotide metabolism (FAC1, adenosine 5 monophosphate deaminase) suggests that such acetaldehyde detoxification might be targeted to the synthesis of precursor for the synthesis of nucleotides. Plants possess metabolic pathways for the *de novo* synthesis of purine nucleotides producing AMP as well as pyrimidine nucleotides producing UMP (Witte and Herde, 2020.) Nucleotides are synthesized from amino acids like Glutamine, Aspartic acid, Glycine, and formyl tetrahydrofolate; the latter is a metabolite related to FDH and formate metabolism (Witte and Herde, 2020).

CYSC1 is co-expressed, under Mo starvation, with 16 proteins whereas it is co-expressed with 11 proteins, under Mo sufficiency (Table 1); interestingly, two glutamate dehydrogenase isoforms, GDH1 and GDH2, are co-expressed with CYSC1, under Mo starvation and Mo sufficiency, respectively, but not under Fe sufficiency or Fe starvation (Supplementary Figure 5). GDHs are enzymes at the branch point between carbon and nitrogen metabolism; both isoforms are localized in mitochondria and GDH2 unless GDH1 is calcium-stimulated (Grzechowiak et al., 2020).

AC4 interacts with tRNAs, and it might be involved in their transport into mitochondria (Hemono et al., 2020); accordingly, *atvdac4* mutant shows severely compromised growth (Hemono et al., 2020). Under Fe starvation, VDAC4 is a hub of two pentatricopeptide-repeat-containing proteins, i.e., PPR596 and At1g60770 (Figure 1). PPR proteins are nuclear-encoded RNA-binding proteins containing repeated motives of approximately 35 aa (Doniwa et al., 2010). PPR596 is involved in the partial C-to-U RNA editing events in the mitochondrial genome and, possibly, also in transcript stabilization and stimulation of translation (Doniwa et al., 2010). Both PPR596 and another PPR protein encoded by At1g60770 also belonging to the VDAC4 co-expression network under Fe starvation (Figure 1) are the closest homologs of the mitochondrial PPR protein PACO1, which affects the flowering time in *A. thaliana* (Emami and Kempken, 2019). VDAC4 is also the hub, under Fe starvation, of L-galactono 1-4 lactone dehydrogenase GLDH, the enzyme that catalyzes the last step in the biosynthesis of ascorbic acid in plants (Wheeler et al., 2015).

VDAC4 is a co-expressed protein of the fumaryl acetoacetate hydrolase (Supplementary Figure 6) and both of them are also hubs in +Fe−Mo and −Fe−Mo PPI networks (Supplementary Table 2). The fumaryl acetoacetate hydrolase catalyzes the formation of fumarate and acetoacetate in the L-tyrosine (Tyr) degradation pathway in plants (Dixon and Edwards, 2006). Altogether, 48 proteins are co-expressed with the fumaryl acetoacetate hydrolase under Fe starvation but none in the other conditions.

## Conclusion

We hereby show how the topological network analysis applied to proteomes obtained from mitochondria purified from plants grown under Fe and/or Mo starvation suggests FDH as a hub of Mo nutrition in agreement with experimental observations (Vigani et al., 2017; Murgia et al., 2020). Such analysis could also suggest other potential hubs for Fe and Mo nutrition, such as VDAC4, CYSC1, ALDH2B7, and fumaryl acetoacetate hydrolase among others, which will be object of our future investigations. VDAC4 and fumaryl acetoacetate hydrolase were found to be hubs by both PPI and protein co-expression networks. The different origin of these network models (basically PPI reconstructed from literature, while co-expression networks from experimental data) strengthens the hypothesis that these two proteins may play a role in Fe and Mo homeostasis, and it confirms the complementarity and synergy of these two approaches in identifying candidate proteins with a role in the processes underlying the investigated phenotypes.

Although the approaches based on computational prediction present some intrinsic limitations, including false-positive interactions or the lack of true ones, they nevertheless promote and support new experimental avenues, including large-scale experimental identification of PPIs, which will improve the effectiveness and accuracy of the proposed approaches.

## Data Availability Statement

The datasets presented in this study can be found in online repositories. The names of the repository/repositories and accession number(s) can be found in the article/Supplementary Material.

## Author Contributions

DD, PMo, GV, and IM conceived the work. DD reconstructed the PPI and co-expression networks with contributions from PMa and SH. IM, GV, and PMo analyzed all the networks and their biological relevance. IM wrote the manuscript with contributions from DD, GV, and PMo. All authors contributed to the article and approved the submitted version.

## Conflict of Interest

The authors declare that the research was conducted in the absence of any commercial or financial relationships that could be construed as a potential conflict of interest.

## Supplementary Material

Supplementary Figure 1 Homology between *C. sativus* and *A. thaliana* proteins.

Supplementary Figure 2 Differentially expressed proteins and *A. thaliana* PPI network modules.

Supplementary Figure 3 Differential FDH correlation degree in co-expression networks.

Supplementary Figure 4 Differential ALDH2B7 correlation degree in co-expression networks.

Supplementary Figure 5 Differential CYSC1 correlation degree in co-expression networks.

Supplementary Figure 6 Differential fumaryl acetoacetate hydrolase correlation degree in co-expression networks.

Supplementary Table 1 PPI hubs in the four nutritional conditions: control (+Fe+Mo), Mo starvation (+Fe−Mo), Fe starvation (−Fe+Mo), Mo and Fe starvation (−Fe−Mo).

Supplementary Table 2 PPI hubs in the four nutritional conditions: Fe sufficiency (consisting in +Mo and −Mo samples), Fe starvation (consisting in +Mo and −Mo samples), Mo sufficiency (consisting in +Fe and −Fe samples), and Mo starvation (consisting in +Fe and −Fe samples).

Supplementary Table 3 Co-expression and physical interaction under Fe sufficiency (+Fe+Mo and +Fe−Mo samples), Mo sufficiency (+Fe+Mo and −Fe+Mo), Fe starvation (−Fe+Mo, −Fe−Mo), and Mo starvation (+Fe−Mo, −Fe−Mo).
