# Supplementary figures and images for: Network Topological Analysis for the Identification of Novel Hubs in Plant Nutrition
Source: Front Plant Sci. 2021 Feb 10;12:629013. doi: 10.3389/fpls.2021.629013 (PMC7928335; doi:10.3389/fpls.2021.629013)

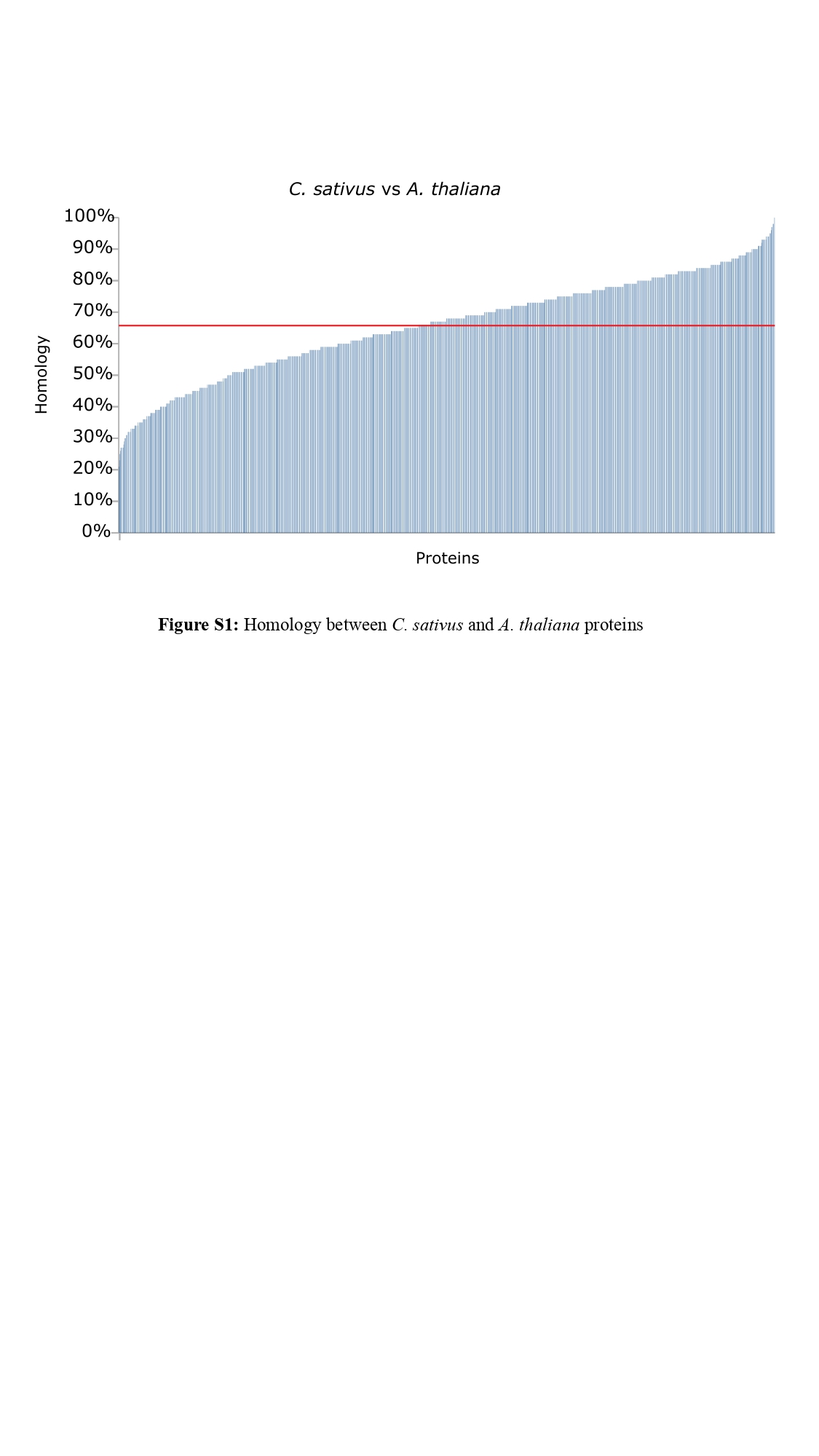

Supplement: Supplementary Figure 1 — Homology between C. sativus and A. thaliana proteins. [file Image_1.JPEG]

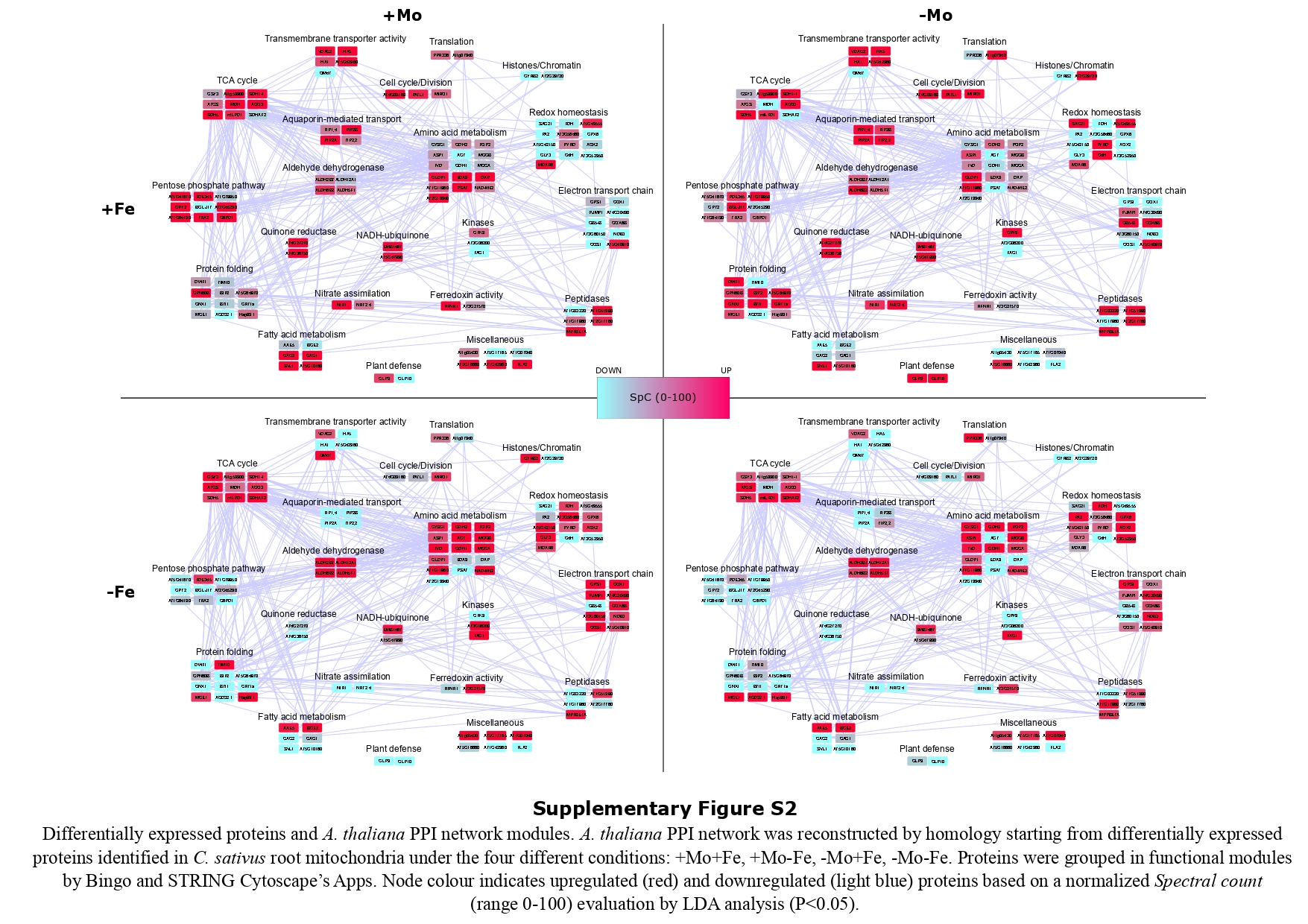

Supplement: Supplementary Figure 2 — Differentially expressed proteins and A. thaliana PPI network modules. [file Image_2.JPEG]

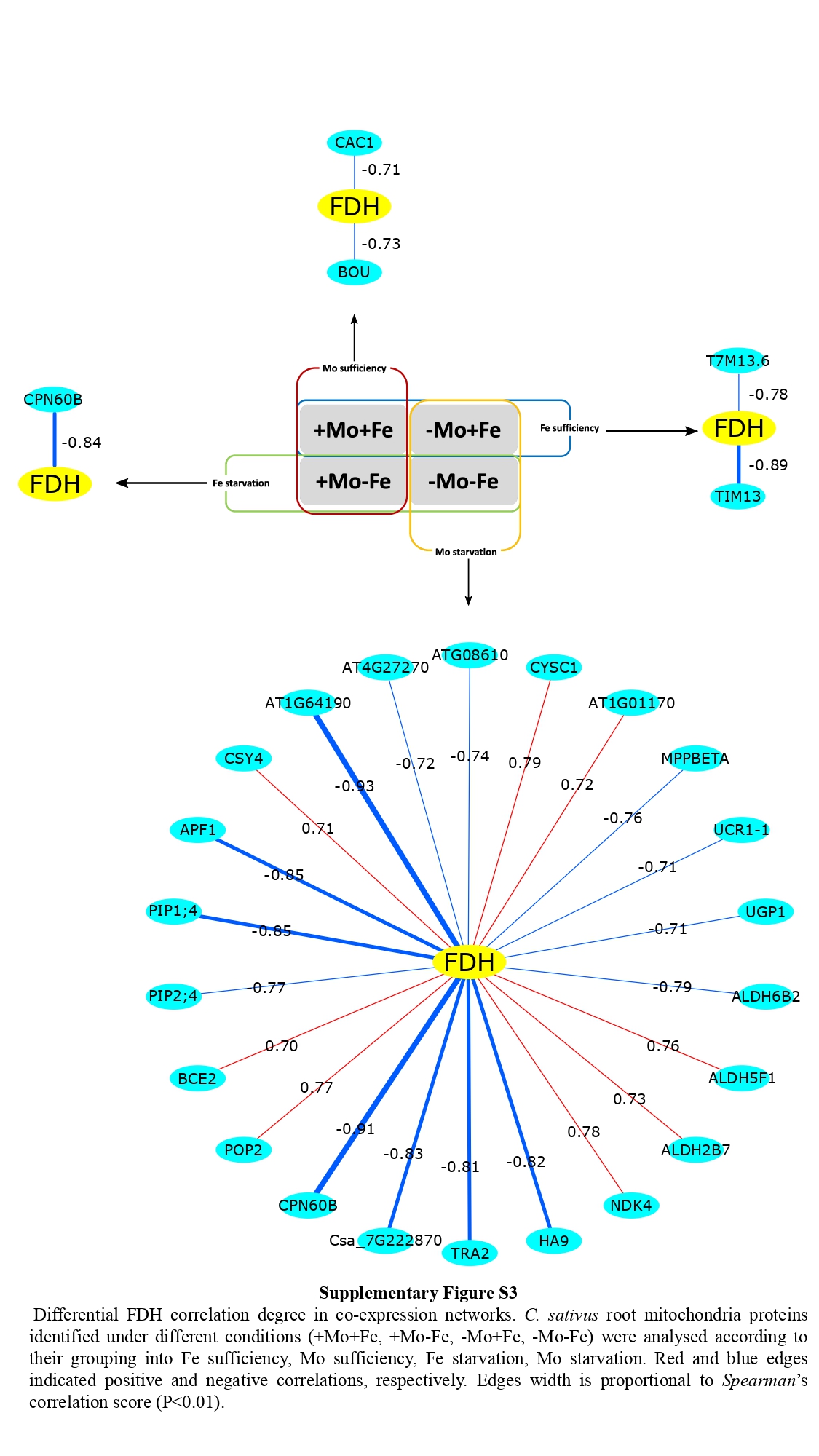

Supplement: Supplementary Figure 3 — Differential FDH correlation degree in co-expression networks. [file Image_3.JPEG]

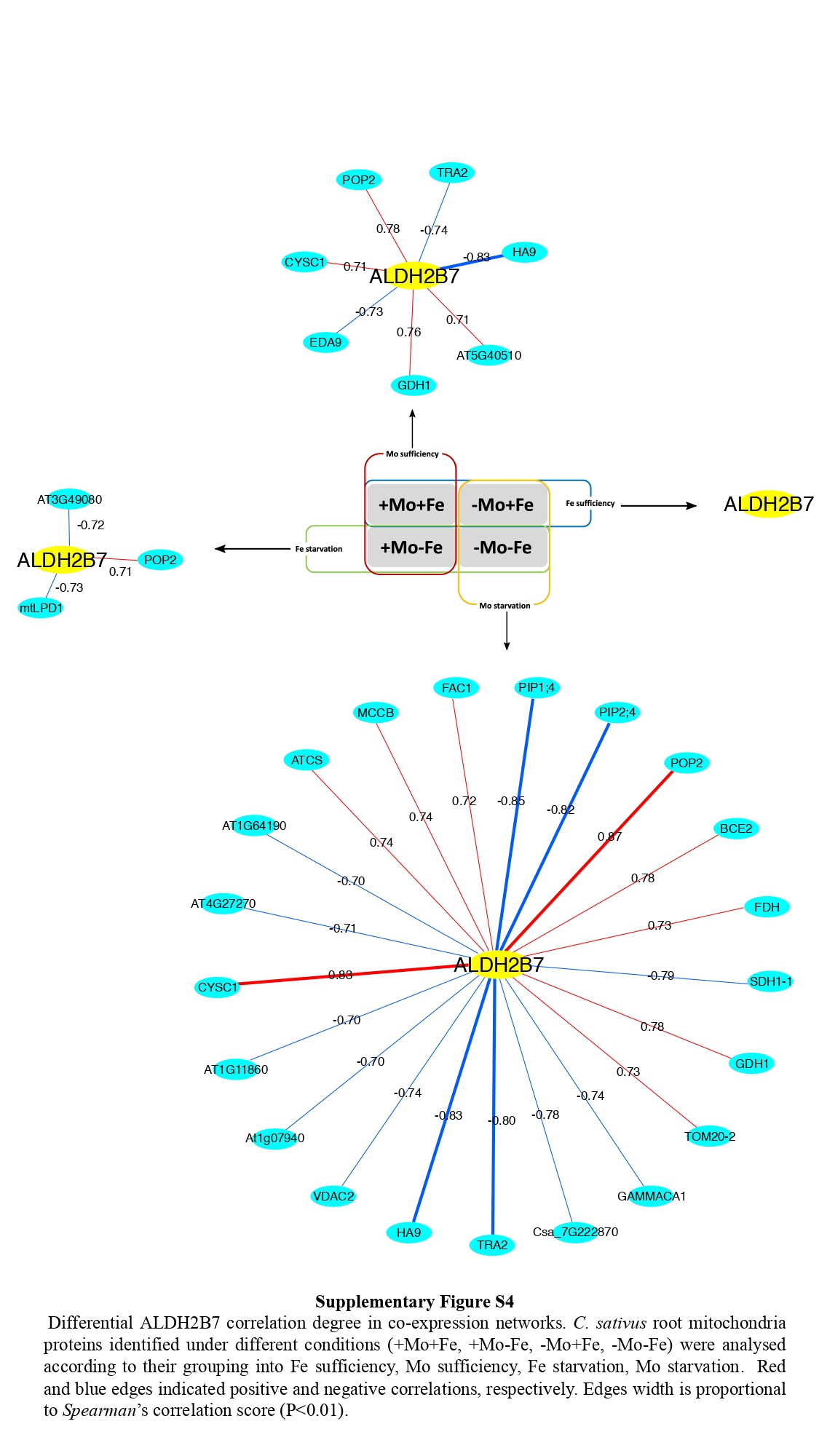

Supplement: Supplementary Figure 4 — Differential ALDH2B7 correlation degree in co-expression networks. [file Image_4.JPEG]

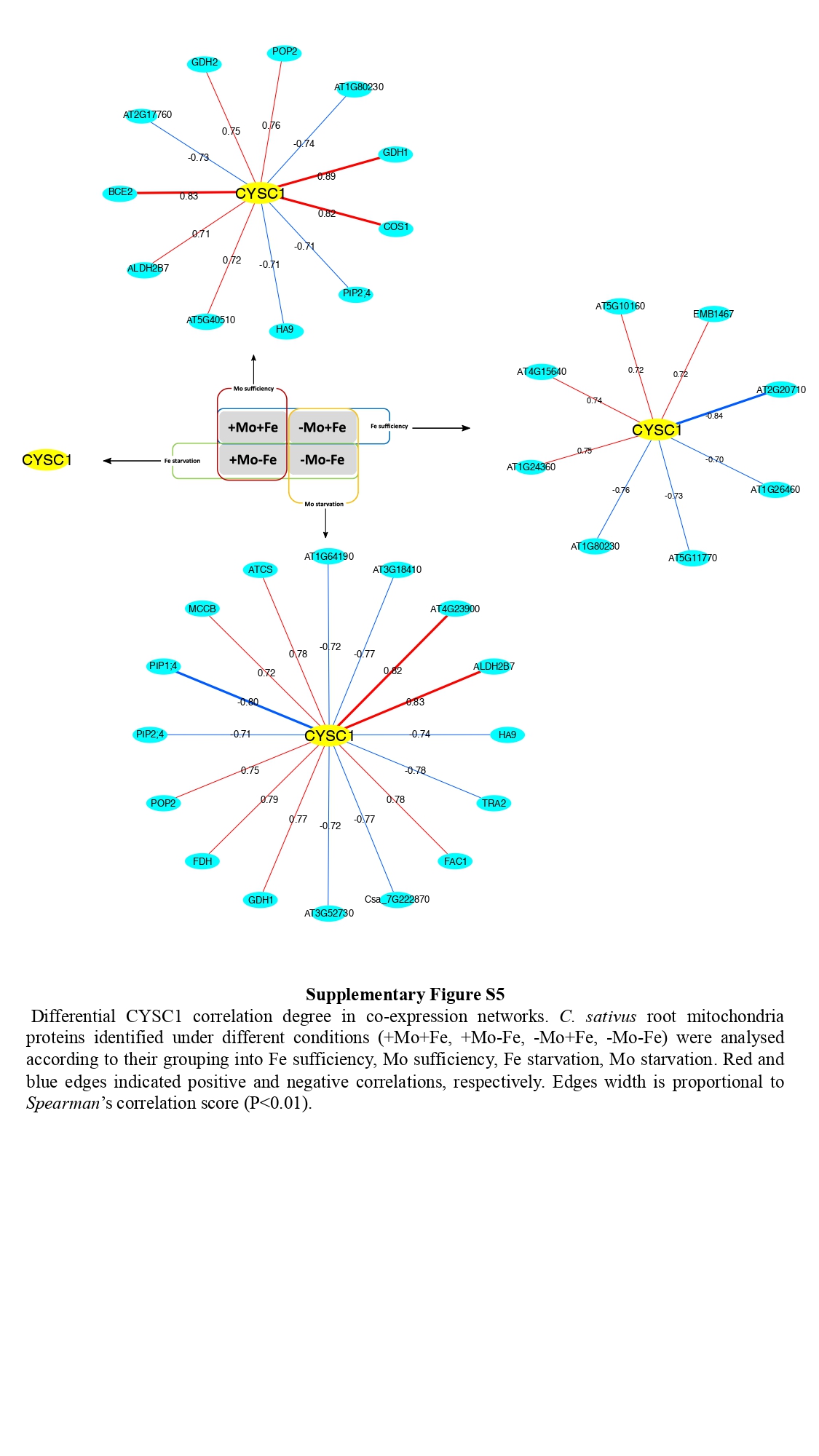

Supplement: Supplementary Figure 5 — Differential CYSC1 correlation degree in co-expression networks. [file Image_5.JPEG]

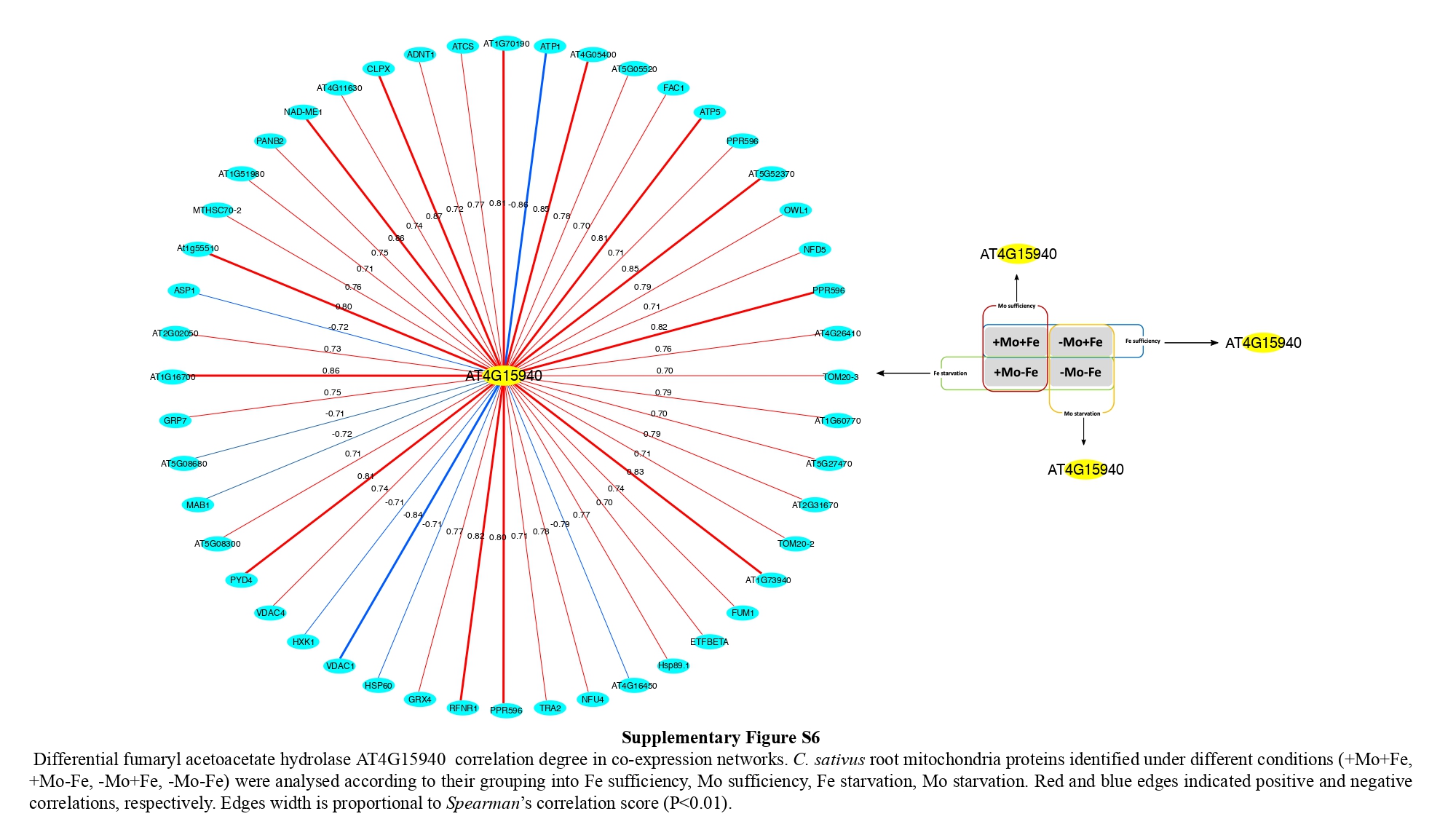

Supplement: Supplementary Figure 6 — Differential fumaryl acetoacetate hydrolase correlation degree in co-expression networks. [file Image_6.JPEG]
